# Flexible alignment in 3D and its applications

**DOI:** 10.1186/1758-2946-3-S1-P30

**Published:** 2011-04-19

**Authors:** A Kalaszi, G Imre, M Vargyas

**Affiliations:** 1 ChemAxon, Budapest, Hungary

## 

Fully flexible 3D alignment method without the need for sampling the conformational space is presented. This approach offers advantages over methods that rely on multiple conformations:

• Higher accuracy is achieved, since unlike discrete sampling it is not prone to missing optimal conformation.

• Smaller database size is needed, pre-calculated conformers are not stored.

The alignment procedure maximizes a score resembles the intersection of the volume of the molecules being aligned. The volume itself can be colored by atomic properties enabling the use of various similarity scores, like shape-, atom type or pharmacophore type similarity.

Similarity scores serve as the basis for 3D virtual screening. However, the calculation of 3D similarities is computationally intensive. In order to achieve high performance fast pre-filtering by 3-dimensional molecular descriptors has been introduced. Intra molecular distance ranges are used as shape descriptors and are pre-calculated by the application of the alignment machinery itself in an initialization stage.

The presentation overviews the mathematical apparatus introduced, elaborates on the implemented methods and presents results.

